# Amyloid-β plaque deposition measured using propagation-based X-ray phase contrast CT imaging

**DOI:** 10.1107/S1600577516004045

**Published:** 2016-04-16

**Authors:** Alberto Astolfo, Aurélien Lathuilière, Vanessa Laversenne, Bernard Schneider, Marco Stampanoni

**Affiliations:** a TOMCAT Beamline, Swiss Light Source, Paul Scherrer Institute, Villigen, Switzerland; b Neurodegenerative Studies Laboratory, Brain Mind Institute, Ecole Polytechnique Fédérale de Lausanne (EPFL), Lausanne 1015, Switzerland; c Institute for Biomedical Engineering, University and ETH Zurich, Zurich, Switzerland

**Keywords:** phase contrast, X-ray CT, brain imaging, amyloid plaques, Alzheimer’s disease

## Abstract

Amyloid beta accumulation into insoluble plaques (Aβp) is known to play a significant role in the pathological process in Alzheimer’s disease (AD). The presence of Aβp is also one of the neuropathological hallmarks for the disease. AD final diagnosis is generally acknowledged after the evaluation of Aβp deposition in the brain. Insoluble Aβp accumulation may also concur to cause AD as postulated in the so-called *amyloid hypothesis*. Therefore, the visualization, evaluation and quantification of Aβp are nowadays the keys for a better understanding of the disease, which may point to a possible cure for AD in the near future. Synchrotron-based X-ray phase contrast (XPC) has been demonstrated as the only imaging method that can retrieve the Aβp signal with high spatial resolution (up to 10 µm), high sensitivity and three-dimensional information at the same time. Although at the moment XPC is suitable for *ex vivo* samples only, it may develop into an alternative to positron emission tomography and magnetic resonance imaging in Aβp imaging. In this contribution the possibility of using synchrotron-based X-ray phase propagation computed tomography to visualize and measure Aβp on mouse brains is presented. A careful setup optimization for this application leads to a significant improvement of spatial resolution (∼1 µm), data acquisition speed (five times faster), X-ray dose (five times lower) and setup complexity, without a substantial loss in sensitivity when compared with the classic implementation of grating-based X-ray interferometry.

## Abstract 2

Synchrotron-based X-ray phase propagation CT is used to visualize and measure amyloid beta accumulation in mouse brains. This technique improves the resolution, the acquisition speed and the X-ray dose if compared with previous methods.

## 1. Introduction   

Alzheimer’s disease (AD) is a chronic neurodegenerative disease, which is the most common cause of dementia. With the global population aging, the prevalence of AD is expected to grow dramatically. Besides the fact that its prevalence significantly affects the economy of nations providing public aid (Reitz *et al.*, 2011 ▸), AD is firstly a serenity disruption for the families which deal daily with an AD-affected relative. There is currently no approved treatment that affects the pathological progression of the disease. The full understanding of the disease process remains unknown. On the other hand, a worldwide research effort constantly refines our understanding of AD. For instance, amyloid beta plaques (Aβp) are insoluble accumulations of protein aggregates that populate the brain tissue of AD patients several years before the occurrence of the first symptoms (Braak & Braak, 1991 ▸; Thal *et al.*, 2002 ▸). Together with neurofibrillary tangles, they are known to be the main neuropathological hallmarks of AD and they are currently used for its definitive diagnosis. Aβp have a size typically between 10 and 120 µm, and they appear as a dense core surrounded by diffuse material. They are normally present in the temporo-parietal neocortex and subcortical nuclei. Although the existence of a relationship between Aβp and AD is established, its role in the disease development is not completely clarified yet. Some interpretations describe the Aβp as a side phenomenon, others as a possible cause of AD as postulated in the so-called *amyloid hypothesis* (Hardy & Selkoe, 2002 ▸).

In parallel to these investigations, there is a need to develop imaging techniques able to proficiently image Aβp deposition in order to establish its role in the disease process. Much progress has been made in this direction. For example, it is now possible to visualize Aβp accumulation *in vivo* using positron emission tomography (PET) combined with specific Aβp tracers such as florbetaben F18 (Neuraceq, Piramal Imaging), florbetapir (AMYViD, Eli Lilly and Company) and flutemetamol (Vizamyl, GE Healthcare) recently approved by the US Food and Drug Administration (http://www.fda.gov). This opened also the possibility of further investigating the deposition of Aβp in AD patients, especially at a preclinical stage (Nordberg *et al.*, 2010 ▸; Doraiswamy *et al.*, 2014 ▸).

At a microscopic level, Aβp become visible using appropriate dye (Saeed & Fine, 1967 ▸) *via* fluorescence microscopy performed on brain sections. The technique provides high resolution and sensitivity with the disadvantage of destroying the sample and partially losing its three-dimensional information.

A recent and interesting alternative to fluorescent microscopy is to use micro-tomography techniques based on synchrotron-based X-ray phase contrast (XPC). It has been demonstrated that Aβp can be correctly visualized during *ex vivo* preclinical studies with a good sensitivity and resolution (Noda-Saita *et al.*, 2006 ▸; Connor *et al.*, 2009 ▸; Pinzer *et al.*, 2012 ▸). Whereas conventional X-ray imaging is based on X-rays attenuation, XPC exploits the phase shift induced by the object on the X-rays. Several applications have confirmed the higher sensitivity of XPC compared with conventional radiology, sometimes opening new applications that were previously impossible using conventional X-ray imaging. To mention some: mammography (Castelli *et al.*, 2011 ▸; Wang *et al.*, 2014 ▸), lung research (Lewis *et al.*, 2005 ▸) and cartilage imaging (Muehleman *et al.*, 2004 ▸; Tanaka *et al.*, 2013 ▸; Horng *et al.*, 2014 ▸). Thanks to XPC, Aβp are visible without the need for contrast agents such as tracers or dyes. Moreover, it makes possible to resolve single Aβp in a preserved three-dimensional geometry. This has the advantage of directly providing information on plaque density, plaque mean diameter, *etc.* These capabilities were proved using the so-called grating interferometer (GI) setup combined with synchrotron radiation (Pinzer *et al.*, 2012 ▸). Although the feasibility of visualising Aβp using a conventional X-ray tube has not been proven yet, the technique has the potential for widespread use, for instance using an XPC technique that can be extended to conventional X-ray tubes (Pfeiffer *et al.*, 2006 ▸; Olivo & Speller, 2007 ▸; Pfeiffer, Kottler *et al.*, 2007 ▸; Hagen *et al.*, 2014 ▸).

However, in all the reported data for Aβp imaging using X-rays, the best resolution achieved is 4 µm using GI (Pinzer *et al.*, 2012 ▸), which is related to the fabrication difficulties of the absorption gratings. As a direct consequence, fluorescence microscopy is still the preferred technique for high-resolution images of Aβp even if it remains limited to two-dimensional images.

Another established XPC technique is X-ray phase propagation (XPP)-based imaging. It is significantly simpler compared with GI as it does not require any additional optical elements between the source and the detector. However, it requires a beam with a very high spatial coherence (Snigirev *et al.*, 1995 ▸; Cloetens *et al.*, 1996 ▸). Experimentally, the phase effects are obtained by moving the detector at a certain distance from the sample in order to allow the X-ray wave refracted by the sample to interfere with the unperturbed one. The result is an enhanced contrast at the boundaries of the sample features that may reveal information from low- absorbing details. Under certain approximations, XPP images can be processed to obtain the sample phase information using phase retrieval algorithms (Paganin *et al.*, 2002 ▸; Moosmann *et al.*, 2010 ▸) translating the edge information into a more convenient area contrast. Paganin’s algorithm (PA) used in the present study assumes the sample has a slowly varying refractive index (Wu *et al.*, 2005 ▸) and it is valid in the near-field regime (Gureyev *et al.*, 2008 ▸). The more these assumptions are not filled the less the phase reconstruction is quantitative (Chen *et al.*, 2013 ▸). For this reason the XPP/PA technique (PP combined with PA phase retrieval) is generally less sensitive although significantly simpler than GI (Diemoz, Bravin & Coan, 2012 ▸; Diemoz, Bravin, Langer & Coan, 2012 ▸). Despite this, XPP/PA has shown interesting capabilities when applied to soft tissues, even if applied outside of its assumption range (Beltran *et al.*, 2011 ▸; Lang *et al.*, 2014 ▸).

In this contribution, we demonstrate that XPP/PA can be optimized to allow three-dimensional visualization of Aβp in a mouse brain. Quantitative results equivalent to GI, such as the total number of Aβp, can be obtained using the same post-processing analysis. A resolution of about 1 µm is achieved without a significant sensitivity loss. Moreover, the technique does not require any scanning of optical elements as in other implementations (Noda-Saita *et al.*, 2006 ▸; Connor *et al.*, 2009 ▸; Pinzer *et al.*, 2012 ▸); it is therefore a faster and simpler technique for non-expert users.

## 2. Material and methods   

### 2.1. Samples preparation   

Animal experiments were performed according to the Swiss legislation and the European Community Council directive (86/609/EEC). Ten brain hemispheres from a ten months old TauPS2APP transgenic AD mouse model were used (Grueninger *et al.*, 2010 ▸). These samples were obtained in another experiment (Lathuilière *et al.*, 2016 ▸) and reused here to limit the use of animals. Some of the mice were treated by immunotherapy to reduce the total amount of Aβp and therefore the samples analyzed showed a broad range of the plaque densities. The mice were sacrificed with an overdose of pentobarbital and perfused transcardially with heparinized PBS with the aim of removing the blood from the animal (including the brain) and to minimize the formation of blood clots. After opening the skull, the brains were dissected and fixed in 4% paraformaldehyde in PBS for 1 h at room temperature. The samples were then transferred in PBS and stored at 4°C until the experiment.

### 2.2. X-ray phase contrast imaging   

**Figure 1:**
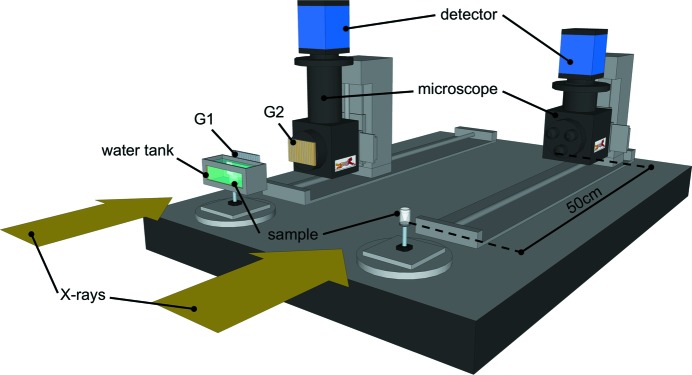
Sketch of the two setups used. The GI one on the left side of the table (with the water tank and the G1/G2 gratings) and the XPP one that requires a propagation distance between the sample and the detector.

All the images presented in this contribution were acquired at the TOMCAT beamline of the Swiss Light Source (Stampanoni *et al.*, 2007 ▸), which is a third-generation synchrotron facility. The ring operates at 2.4 GeV with an electron beam of 400 mA kept stable (∼0.5%) with the so-called top-up mode. The beamline is equipped with a multilayer monochromator (W/Si) with a bandwidth of 1%. Two different setups commonly available at TOMCAT were used (as schematized in Fig. 1 ▸) and compared.

The first was a gratings interferometer (GI), which consists of two gratings, a silicon phase grating (called G1) with a pitch of 3.98 µm and a gold absorption grating (called G2) with a pitch of 2 µm. The system is designed for an X-ray energy of 25 keV and it is almost identical to the one described by Pinzer *et al.* (2012 ▸) that we consider our gold-standard for Aβp visualization using X-rays. In brief, the samples were placed in a cylindrical plastic container (14 mm in diameter) placed 28 m from the source. Three half-brain samples were placed in the container, which covered the system field of view (FOV). The samples were kept wet in degassed PBS. The container was then immersed into an aquarium filled with degassed demineralized water and it was free to rotate without any leaking. PBS preserves the sample from unwanted shrinking during the scan, which would translate in motion artifacts on the computed tomography (CT) reconstruction. The aquarium allows the exposure time to be optimized and it prevents the phase-wrapping artifacts caused by the interface air-container. The degassing process decreases significantly the formation of gas bubbles during the scan, which would introduce additional artifacts. G1 was placed as close as possible to the aquarium and G2 was aligned 121 mm downstream of it (third Talbot fractional distance). Behind G2, a 350 µm-thick LuAG:Ce scintillator converted the X-rays to optical light, which was read by a CMOS camera (PCO.Edge, PCO AG, Germany) through two lenses (NA = 0.2). The detector has a 2560 × 2160 array of 6.5 µm × 6.5 µm pixels. The resulting FOV of 16.6 mm × 4.16 mm (the vertical dimension is limited by the beam height) was enough to scan three samples in a single CT scan consisting in three vertical scans of 4.16 mm height. A total of 1440 projections were collected over 180° for seven positions of G1 over the 2 µm period, with an exposure time per projection of 200 ms. Standard Fourier analysis for GI (Momose *et al.*, 2004 ▸) was applied to the images to retrieve the δ map of the brains.

The second setup was optimized for XPP/PA in terms of distances and detector resolution. As in GI, the samples were kept in PBS but this time the aquarium was not used because the artifacts were not compromising the final result. The samples were placed 50 cm upstream of the scintillator. The same CMOS camera used for GI was coupled to an Optique Peter microscope with 2× magnification (NA = 0.08) that translates to an effective pixel size of 3.25 µm. The scintillator used was a 100 µm-thick LuAG:Ce. The higher magnification, compared with the GI setup, was necessary to be able to detect the edge-enhancement signal produced by the sample. The FOV became 8.3 mm × 4 mm, therefore the scan was limited to one sample per time fully acquired in three vertical scans of 4 mm height. A total of 1440 projections were acquired while the sample was rotated continuously over a range of 180°, together with 100 flat images (without the sample) and ten dark images (without the beam), with an exposure time of 200 ms. The two setups used are depicted (in a simplified version) in Fig. 1 ▸. The main acquisition parameters are summarized in Table 1 ▸.

During the same XPP experiment, 4× and 10× objectives were used to demonstrate the capability of increasing the imaging resolution using a similar setup. The camera pixel sizes were then 1.6 µm and 0.65 µm, which translates into a FOV of 4.2 mm × 3.5 mm and 1.7 mm × 1.4 mm, respectively. The scintillator was a LAG:Ce crystal of 20 µm thickness. The switch to higher resolution is particularly simple at TOMCAT beamline, where the Optique Peter microscope is equipped with a revolver, which accommodates three interchangeable objectives. In these two cases the sample–detector distance was reduced to 20 and 10 cm, respectively.

### 2.3. Image post-processing   

GI images were analyzed following the method proposed by Pinzer and colleagues (Pinzer *et al.*, 2012 ▸). In brief, the Fourier analysis for GI is used to retrieve the differential phase contrast projections out of the so-called phase stepping images obtained by moving G1. Standard sinograms were then created and normalized with a Gaussian curve to avoid the background inhomogeneity due to the monochromator instability. The δ three-dimensional reconstructions are then performed using a gridding algorithm (O’Sullivan, 1985 ▸) implemented on the TOMCAT cluster for fast computing (Marone & Stampanoni, 2012 ▸). Manual segmentation of the cortex was performed using the *ImageJ* plug-in ‘Segmentation Editor’ trying to be as consistent as possible between the samples. In order to decrease the noise level and to enhance the signal from Aβp, a ‘three-dimensional LoG’ filter (2^3^ voxels size), which is freely available as an *ImageJ* plug-in (Sage *et al.*, 2005 ▸), was applied over the reconstructed volume. Looking carefully at the images, a threshold was selected to discriminate the plaques from the background. Then the plaques were counted on the segmented cortex using *IDL* (Exelis, USA) standard routines. To minimize the noise contribution to the total number of plaques we excluded from the total count the Aβp smaller than ten voxels. As the threshold choice is partially arbitrary, we decided to repeat the count over an interval of five values ranging from the selected threshold plus/minus the background noise present on the LoG3D slices, which is similar to the method reported in another contribution (Astolfo *et al.*, 2013 ▸). The final result used for comparison is the mean ± standard deviation calculated over these five measures.

A parallel analysis was run on the XPP data. The projections were first processed using the phase retrieval PA. An optimization of the δ/β ratio was run in order to maximize the contrast between the brain tissue and the Aβp and the best ratio found was δ/β = 300. The reconstructed slices (acquired with higher resolution compared with the GI data) were digitally resized after the three-dimensional reconstruction in order to match the same pixel size for a fair comparison. The relationship between the reconstructed δ and the image greyscale is not the same for the two techniques used. Therefore, given that the final image gray values are significantly different between the two techniques, the threshold to choose for counting the plaques is a delicate issue that could introduce an offset on the final results. To mitigate this effect, we decided to use the same strategy used with GI data (carefully choose a reasonable value) with the additional step of calibrating the threshold for one representative sample (ID33 in Fig. 4) with the result obtained with GI. This value has been used then for all the other nine samples.

The images at higher resolution were reconstructed using the PA without any additional post-processing.

## 3. Results   

**Figure 2:**
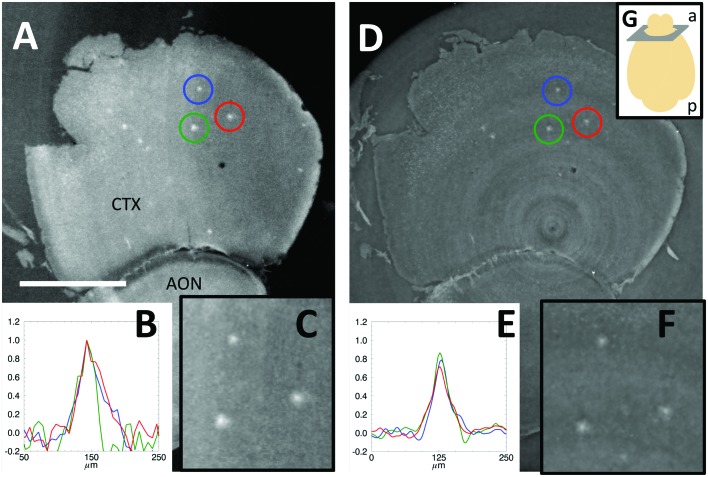
Phase signal reconstructed from (A) GI and (D) XPP/PA of the cortex (CTX) and anterior olfactory nucleus (AON). Horizontal profiles of the plaques marked with the coloured circles normalized to the GI ones (B, E). Zoom of the analyzed plaques (C, F). Sketch showing the image position and orientation according to the anterior (a) and posterior (p) parts of the brain (G). Scale bar 1 mm.

As an example of the images obtained using the GI and XPP setups, Fig. 2 ▸ shows the δ reconstructed map of approximately the same region of a mouse brain sample presenting several Aβp (maximum intensity projection over 32.5 µm-thick slice) obtained with the two techniques. As a first qualitative result, the Aβp are quite visible with XPP/PA. Looking more carefully, the limitations of XPP/PA become clearer. The greyscale value between the tissue and the PBS (in which the brain is immersed) is notably different in GI, whereas in XPP/PA it is almost similar. This finding is expected because GI is known to be a quantitative technique (Herzen *et al.*, 2009 ▸) over a wider range of δ values when compared with XPP/PA. The latter technique mainly provides a qualitative reconstruction if the algorithm model assumptions are not fulfilled (Chen *et al.*, 2013 ▸). This difference is also a direct consequence of the parameter optimization for the visualization of Aβp. Despite the lack of XPP/PA accuracy, it remains possible to identify the Aβp for the purpose of counting them, for which a relative δ difference is important. In order to provide a more detailed comparison between the two techniques, three intensity profiles corresponding to three relatively large Aβp are shown in Figs. 2B and 2E ▸. The intensities were normalized to the ones measured by GI. The contrasts of XPP/PA-reconstructed plaques are approximately 20% lower than using GI. The background noise is visibly lower in XPP/PA because of the low-pass spatial frequency filter nature of the PA (Paganin *et al.*, 2002 ▸).

**Figure 3:**
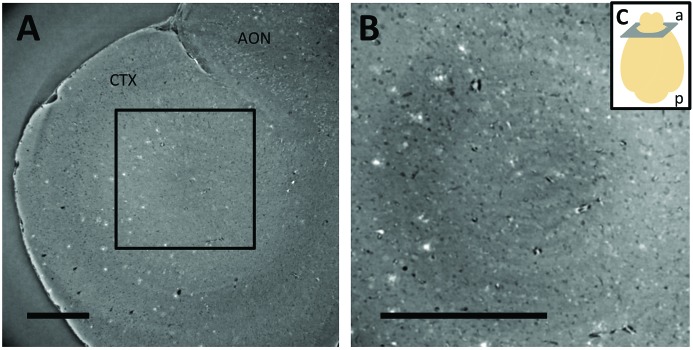
Phase reconstructed slices of the same brain sample obtained at higher resolution using PP, with a pixel size of 1.6 µm (A) and 0.65 µm (B). The image shows part of the cortex (CTX) and anterior olfactory nucleus (AON). The region of interest outlined in A is shown in B. Sketch showing the image position and orientation according to the anterior (a) and posterior (p) parts of the brain (C). Scale bar 500 µm.

In Fig. 3 ▸, we show that the XPP/PA setup allows increasing the resolution on Aβp imaging without the limitation of the G2 gratings pitch as in GI. Depending on the experimental needs in terms of FOV and resolution, it is possible to visualize Aβp with a pixel size of 1.625 µm (∼4.3 mm × 3.5 mm of FOV, Fig. 3A ▸) and with 0.65 µm (∼1.7 mm × 1.4 mm of FOV, Fig. 3B ▸). Moreover, higher resolutions could be achieved using the 20× and 40× magnification objectives available on the same microscope at the TOMCAT beamline. However, the reduced FOV (416 µm × 351 µm using the 40× objective) limits the investigation to a very small volume, allowing for only local visualization of the distribution of Aβp in the brain.

It is not trivial to provide a fair comparison between these two techniques for measuring the total number of plaques per sample. In the two approaches, the measured signal derives from the X-ray phase shift induced by the Aβp. However the signal detection, processing and visualization are distinct. This induces differences in image noise propagation, contrast and artifacts, among others. Moreover, the manual segmentation of the cortex introduces unavoidable systematic errors. For instance, sample manipulation could lead to the deterioration of some areas (all the samples were first scanned with the GI and then with in the XPP mode); some reconstruction ring artifacts may cover different parts of the brain which may lead to a bias in the measured density of Aβp*.* In these situations, the affected areas have been excluded before segmentation. Although these effects may inevitably alter the absolute number of Aβp, we obtained a satisfying level of correlation between the values measured with each of these two techniques.

**Figure 4:**
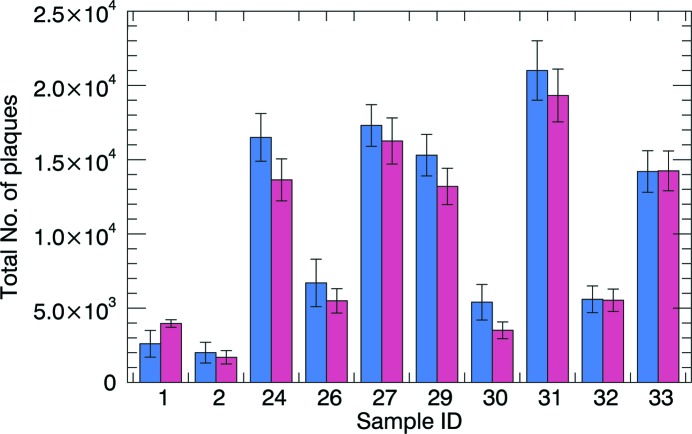
Total number of plaques measured with GI (blue) and XPP/PA (red) in ten individual samples.

The graph in Fig. 4 ▸ shows the comparison of the total number of Aβp measured with the two techniques. Using a Student’s *t*-test (*tm_test* function, *IDL*) for statistical comparison between nine pairs of samples (excluding the sample used for threshold calibration), we calculated a level of significance above 1%. Therefore they do not have different means.

**Figure 5:**
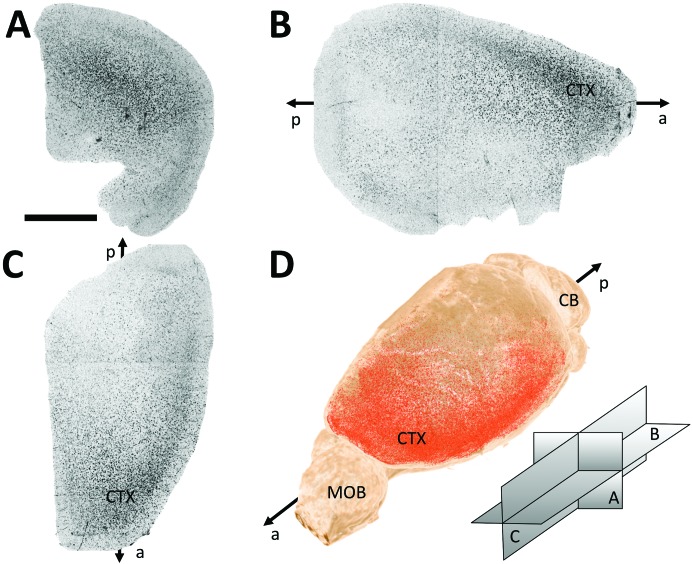
Projections (coronal A, axial B and sagittal C; orientation scheme shown in D) of the plaque signals (black trace) in a representative three-dimensional brain sample. Three-dimensional rendering with the segmented plaques in red (D). Cortex (CTX), cerebellum (CB) and main olfactory bulb (MOB) regions are indicated along the antero-posterior axis of the brain. Scale bar 2 mm.

The three-dimensional information obtained from these measurements allows for various methods of data analysis. As an example, Fig. 5 ▸ shows the minimum intensity projections (MIPs) of the Aβp signal from one representative sample (views 5A, 5B and 5C ▸). MIPs can be interpreted as thick histology slices containing information from the whole sample. They can be useful for a quick comparison over several samples. Those images contain qualitative information of the total number of Aβp, their density and location. A three-dimensional rendering can be also useful (Fig. 5D ▸ and supplementary video) as it permits a detailed study of the exact location of the accumulation of Aβp (Limaye, 2012 ▸).

## 4. Discussion   

The capability to visualize Aβp by means of XPC techniques has been demonstrated and exploited in the past few years (Connor *et al.*, 2009 ▸; Pinzer *et al.*, 2012 ▸; Lathuilière *et al.*, 2016 ▸), using crystal-analyzer-based imaging and GI. XPC is an interesting approach because it provides three-dimensional information of Aβp combined with a relatively high resolution which no other technique can do. Pinzer *et al.* used GI to show the possibility to retrieve, not only a good resolution three-dimensional map of Aβp for entire mouse brains, but also how to use the data for quantitative measures of the Aβp (*e.g.* total number, density, *etc.*), comparing the results with histology and fluorescence microscopy. In all the previously published experiments of XPC techniques applied to imaging of Aβp, synchrotron radiation was required together with some additional imaging components (an analyzer crystal or two gratings). Moreover, multiple images were acquired to retrieve the phase information. These two techniques are not readily available for general use. This limits the possibility for a wide use of XPC in the context of Alzheimer’s disease research, despite obvious advantages for the quantification of Aβp. Moreover, complicated imaging setups translate in higher costs in terms of equipment, its service and its maintenance. Finally, the use of multiple image techniques requires high stability of setup components, generating new sources of possible experimental artifacts.

In the present report, we demonstrate and exploit XPP/PA as an alternative XPC technique for Aβp imaging. This approach is experimentally simpler compared with the previously proposed techniques. We compare XPP/PA with GI, as the latter technique can arguably be considered as the gold standard for this application. This comparison highlights some advantages of XPP/PA over GI. Notably, XPP does not require any additional optical elements, resulting in a simple setup and it does not need precise alignment. Second, the use of a single-image approach (instead of scanning the grating or the analyzer crystal) decreases the requirements for stability. Moreover, the use of single images per angular step allows the reduction of the X-ray dose applied to the sample, as well as the total acquisition time. In the presented setup, we have reduced by fivefold the acquisition time per sample compared with GI, with a fivefold lower X-ray entrance dose (taking into account the radiation absorbed by the water in the aquarium) and we have been able to obtain images with higher resolution. As a trade-off, the contrast is decreased by about 20% (Fig. 2 ▸), however without compromising the detection of Aβp.

One drawback of GI compared with fluorescence microscopy is the lower resolution achievable. At the moment, GI resolution is limited by the gratings fabrication and, in the case of Aβp, it has never been better than 4 µm (Pinzer *et al.*, 2012 ▸). XPP/PA is not affected by this limitation. The images in Fig. 3 ▸ show Aβp acquired with a pixel size up to 0.65 µm in three dimensions. Further improvements are also possible, if the FOV reduction is not crucial. Finally, the quantification of the Aβp shows a good agreement between GI and XPP demonstrating that the new technique can be used as well as GI for assessing Aβp in the context of Alzheimer’s disease research (Fig. 4 ▸).

Of note, a ‘classic’ implementation of GI has been recently carried out, with further developments of the technique. Several beamlines worldwide do offer GI capabilities or are working on such systems: *e.g.* ID19 at ESRF, France (Pfeiffer, Bunk *et al.*, 2007 ▸); 20XU at SPring-8, Japan (Momose *et al.*, 2006 ▸); BL13W1 at SSRF, China (Chen *et al.*, 2014 ▸); Elettra, Italy; SOLEIL, France; Diamond, UK; CLS, Canada; Australian Synchrotron, Australia; APS, USA). On the other hand, XPP is potentially available with no special efforts in all synchrotron facilities with X-ray imaging beamlines. Therefore, XPP/PA will be broadly accessible to potential users for the imaging of Aβp.

## Supplementary Material

of the 3D rendering of the plaque signals in a representative 3D brain sample. DOI: 10.1107/S1600577516004045/mo5134sup1.avi

## Figures and Tables

**Table 1:** Summary of the acquisition parameters for GI and XPP imaging

	Grating interferometer	Phase propagation
Energy (keV)	25	25
Photon flux density (photons mm^−2^ s^−1^)†	1.37 × 10^11^	1.9 × 10^11^
Pixel size (µm)	6.5 × 6.5	3.25 × 3.25
FOV (mm)	14 × 4	8.32 × 4
Projections	1440 × 7	1440
Total entrance dose (Gy)	∼30000	∼5800
Scintillator	LAG:Ce 350 µm	LAG:Ce 100 µm
Numerical aperture	0.2	0.08
Exposure time (ms)	200	200
Total acquisition	∼100 min per half brain	∼20 min per half brain

† Flux measured with a calibrated diode at sample position with precisions lower than a few percent (Lovric, 2015 ▸).
